# ALFY-Controlled DVL3 Autophagy Regulates Wnt Signaling, Determining Human Brain Size

**DOI:** 10.1371/journal.pgen.1005919

**Published:** 2016-03-23

**Authors:** Rotem Kadir, Tamar Harel, Barak Markus, Yonatan Perez, Anna Bakhrat, Idan Cohen, Michael Volodarsky, Miora Feintsein-Linial, Elana Chervinski, Joel Zlotogora, Sara Sivan, Ramon Y. Birnbaum, Uri Abdu, Stavit Shalev, Ohad S. Birk

**Affiliations:** 1 The Morris Kahn Laboratory of Human Genetics, National Institute for Biotechnology in the Negev and Faculty of Health Sciences, Ben Gurion University, Beer Sheva, Israel; 2 Department of Life Sciences, Ben Gurion University, Beer Sheva, Israel; 3 Genetics Institute, HaEmek Medical Center, Afula, Israel; 4 Department of Community Genetics, Public Health Services, Ministry of Health, Jerusalem, Israel; 5 Genetics Institute, Soroka University Medical Center, Ben Gurion University, Beer Sheva, Israel; Max Planck Institute for Molecular Genetics, GERMANY

## Abstract

Primary microcephaly is a congenital neurodevelopmental disorder of reduced head circumference and brain volume, with fewer neurons in the cortex of the developing brain due to premature transition between symmetrical and asymmetrical cellular division of the neuronal stem cell layer during neurogenesis. We now show through linkage analysis and whole exome sequencing, that a dominant mutation in ALFY, encoding an autophagy scaffold protein, causes human primary microcephaly. We demonstrate the dominant effect of the mutation in drosophila: transgenic flies harboring the human mutant allele display small brain volume, recapitulating the disease phenotype. Moreover, eye-specific expression of human mutant ALFY causes rough eye phenotype. In molecular terms, we demonstrate that normally ALFY attenuates the canonical Wnt signaling pathway via autophagy-dependent removal specifically of aggregates of DVL3 and not of Dvl1 or Dvl2. Thus, autophagic attenuation of Wnt signaling through removal of Dvl3 aggregates by ALFY acts in determining human brain size.

## Author Summary

One of the major events in human evolution is the significant increase in brain volume in the transition from primates to humans. The molecular pathways determining the larger size of the human brain are not fully understood. Hereditary primary microcephaly, a neurodevelopmental disorder in which infants are born with small head circumference and reduced brain volume with intellectual disability, offers insights to the embryonic molecular pathways determining human brain size. Previous studies have shown that human microcephaly can be caused by mutations in genes affecting cell division processes, such as cell cycle regulation, DNA replication, primary cilia formation and centriole and centrosome duplication. We now show a novel molecular pathway determining human brain size: human primary microcephaly can be caused by a mutation in ALFY, a gene that encodes an autophagy scaffold protein. In fact, transgenic flies over expressing the mutant form of human ALFY recapitulate the human disease phenotype of microcephaly. We show the molecular pathway through which ALFY regulates cell division and differentiation: we demonstrate that ALFY normally controls removal of aggregate of DVL3, and through this regulates Wnt signaling, a major molecular pathway in embryogenesis. Thus, Wnt signaling, controlled by ALFY-mediated aggregate removal of DVL3, determines human brain size and human microcephaly.

## Introduction

Primary microcephaly has mostly been reported as an autosomal recessive trait coupled with mild to severe intellectual deficit [1, 2]. The developing brain of higher mammals begins with a pseudostratified layer of apical neuroepithelial (NE) progenitor (AP) cells, which are attached to the apical and pial surfaces maintaining their polarity. At the onset of neurogenesis, NE cells turn into radial glial cells (RGCs) that will generate, directly or indirectly, all neurons. The RGCs undergo self-renewing cell divisions, later switching from symmetric to asymmetric divisions, giving rise to RGC daughter cells and differentiating basal progenitor (BP) cells which maintain their proliferative state and will later differentiate into neuronal cells [3, 4].

The number of proliferative division rounds of both APs and BPs prior to their differentiative division is critical for establishing proper brain size and development [3, 5, 6]. Therefore, it is not surprising that most genes known to date to be associated with MCPH are involved in the processes of mitosis, cell cycle regulation, DNA replication and primary cilia formation and stabilization. It is believed that premature transition between symmetrical to asymmetrical divisions during brain development is the main cause for primary microcephaly [5–7]. This premature transition results in an insufficient number of precursor cells within the neuronal stem cell (NSC) population, and eventually leads to reduced number of neurons in the cortex [5]. To date, 16 loci and genes have been associated with autosomal recessive primary microcephaly (MCPH), [5, 8–13] and two genes, *KIF11* [14] and *DYRK1A* [15], have been linked to autosomal dominant primary microcephaly. Most of the known MCPH genes are expressed predominantly in neuronal tissues during embryonic development and have been implicated in neuronal differentiation [5, 9, 14, 15]. We now demonstrate that autosomal dominant primary microcephaly can be caused by a dominant mutation in *ALFY*, encoding a master scaffold protein which facilitates removal of aggregated intracellular proteins. Through Drosophila and in-vitro experiments, we show that ALFY controls Wnt signaling by regulating DVL3 aggregation, likely in an autophagy-dependent manner, unveiling novel molecular pathways of normal brain development and primary microcephaly.

## Results

### Genetic analysis

**Fig 1:**
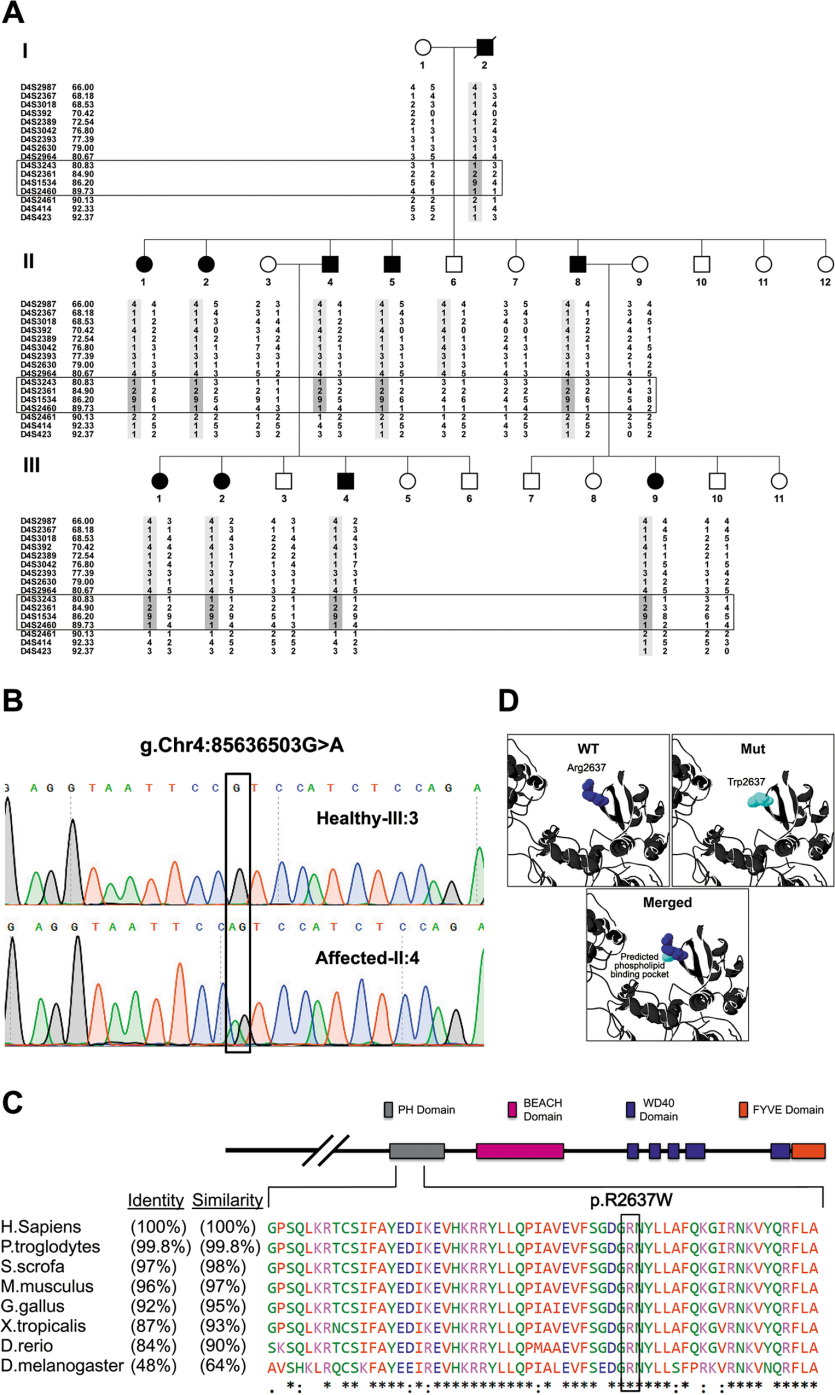
Pedigree of studied kindred and the *ALFY* mutation. **A. The affected Arab Israeli kindred and fine mapping:** Haplotype shared by affected individuals is highlighted in light gray shading. Note that the haplotype of healthy individual II:6 determines the minimal shared ~9 Mbp disease-associated locus (rectangular black box) between markers D4S3243 and D4S2460. Haplotypes for individual I:2 (DNA not available) were reconstructed using data of two successive generations. Marker positions are given in Mbp. **B. The ALFY g.Chr4:85636503G>A, c.7909C>T, p.R2637W mutation.** Sanger sequencing of healthy (III:3) and affected (II:4) individuals. **C. Conservation of *ALFY* throughout evolution.** Substituted Arginine (black box) is extremely conserved and is located within a conserved PH domain. **D. Structural prediction of the mutated ALFY PH-BEACH domain based on the solved structure of PH-BEACH domain of neurobeachin**. Modeling suggests that the Arginine residue protrudes to the predicted phospholipid binding pockets and is presumably critical for the domain function.

A large kindred presented with apparently autosomal dominant isolated primary microcephaly with mild to moderate intellectual disability (Fig 1A). Head circumference of all affected individuals was <3 standard deviations below the mean per age. None of the patients had apparent dysmorphic features or ocular malformations, and thorough physical examination revealed no further abnormalities or failure to thrive. Magnetic resonance imaging (MRI) demonstrated microcephaly with no structural defects. Genome-wide linkage analysis followed by fine mapping identified a ~9 Mb haplotype on chromosome 4, which was shared by and unique to the affected individuals in the kindred (Fig 1A). Maximal LOD score was 3.44 at D4S1534 (θ = 0). Within the 9 Mb locus, whole exome sequencing for individual II:4 (Fig 1A) identified no homozygous mutations and only 2 heterozygous variations: in *ALFY* (termed also *WDFY3*) and in *CXCL11*. While the *CXCL11* variation was found in 4 of 200 Israeli Arab healthy controls, none of the controls had the *ALFY* variation. The *ALFY* variation segregated within the kindred as expected. Thus, the only variation common and unique to the affected individuals of the kindred was a missense mutation in *ALFY*: c.7909C>T g.Chr4:85636503G>A, NM_014991.4, p.R2637W (Fig 1B).

### ALFY conservation

The missense mutation, within an extremely conserved residue (Fig 1C) of the PH-BEACH domain, results in substitution of a hydrophilic positively charged arginine to aromatic hydrophobic tryptophan. Reconstruction modeling based on the resolved 3D structure of the PH-BEACH domain of neurobeachin, [16] which has high homology with the PH-BEACH domain of ALFY (49% identity, 65% similarity over 265 amino acids of the PH-BEACH domain), predicted that the arginine at position 2637 normally protrudes into a putative binding pocket. Although phospholipid binding by the PH-BEACH domain of ALFY has never been demonstrated, it is thought that such binding might occur at this site, based on known function of PH domains [17]. Replacement of arginine with tryptophan at this position is predicted to dramatically alter the putative binding domain function (Fig 1D).

ALFY is a nuclear protein, which upon accumulation of protein aggregates in the cell shuttles to the cytoplasm, where it serves as a scaffold protein facilitating autophagy-mediated removal of such cytosolic protein aggregates [18–21]. The 1200 amino acid C-terminal of the 3526 amino acid long ALFY is sufficient for these functions, in line with the putative functional domains (PH-BEACH, WD40 and FYVE) within this fragment [18–21]. ALFY is extremely conserved throughout evolution, and its conservation is highest at the C-terminal (Fig 1C). Its homology with its Drosophila ortholog Bluecheese (*Bchs*) is relatively high (48% identity and 64% similarity in full protein sequence), and extremely high surrounding the mutation (Fig 1C).

### In-vivo Drosophila experiments

**Fig 2:**
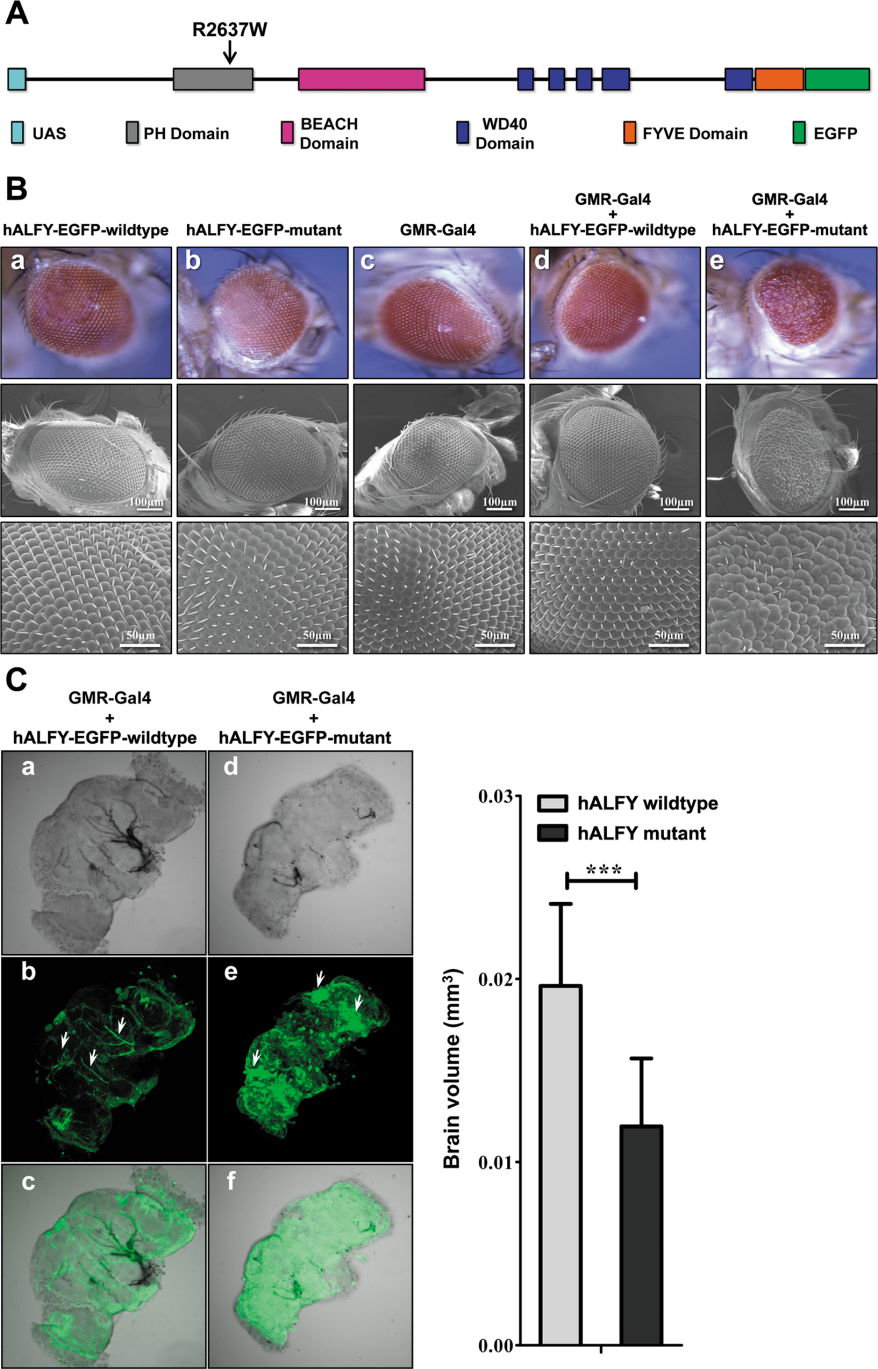
Mutant *hALFY* causes dominant phenotypes in Drosophila. **A**. **Cloning construct for transgenic flies experiments.** The C Terminal part, containing all functional domains of human *ALFY*, was cloned in-frame with an EGFP sequence at the 3' end to form a fusion protein. An upstream activating sequence (UAS) was added at the 5' end of the sequence for controlled expression. **B. Eye phenotype.** Expression of both wild type (WT) and mutant *hALFY* alleles was driven with the GMR-GAL4 promoter. Fly eyes were analyzed in terms of pigmentation, size, shape and surface texture, and representative digital eye images were taken. Higher resolution images (SEM) obtained at X400 and X1200 magnification. While all original hALFY wild type, mutant and GMR driver lines, as well as the GMR hALFY wild type driven expression resulted in normal eye development (a-d), the GMR mutant hALFY driven expression resulted in a severe eye phenotype (e). Mutant eyes are disorganized, and malformed with abnormal omatids formation and orientation. Omatids appear to be fused and bristle morphology and distribution is abrupted with missing or fused bristles. **C. Brain phenotype.** Brains of flies of actin-driven expression of wild type *hALFY*-EGFP (a-c) and mutant *hALFY*-EGFP (e-f). Brains of wild type and mutant flies were dissected and visualized with confocal microscope in bright field (a, d) and 488nm filter (b, e) at X20 magnification. As opposed to the wild type allele, homogenous actin-driven expression of the mutant allele resulted in the formation of smaller, less organized and denser brains (a, d). Mutant brains were 40–60% smaller in volume than wild type (t test two tailed analysis, wild type n = 18, Mut n = 31, P_value_<0.0001). Expression profiling using the EGFP tag, clearly demonstrates that the mutant protein tends to accumulate in the brains of mutant flies in large clusters of cells (e–white arrows) as opposed to the expression pattern in the wild type (b–white arrows). Mutant cells are dispersed throughout the brain and appear to be smaller in size, less elongated and greater in number than the wild type.

To explore the functional effect of the human *ALFY (hALFY)* mutation, we utilized the Drosophila model system, since *ALFY* shares high degree of similarity with its fly ortholog *bchs* [22, 23]. In line with the dominant heredity of the human phenotype, we generated transgenic flies over-expressing EGFP-tagged wild type and mutant forms of the 1226 amino acids of the C-terminal of hALFY under the control of the upstream activating sequence (UAS) Gal4 system, allowing tissue-specific expression. While ubiquitous expression of the wild type hALFY C-terminal construct under the control of actin promoter resulted in no abnormal phenotype, in flies ubiquitously expressing the human mutant allele, the larvae appeared to develop normally and were able to reach pupae stage but did not eclose. All pharates of the mutant *hALFY* allele were similar to the wild type ones in terms of size and morphology. To study possible effects of the *hALFY* mutation on Drosophila brain development, we micro-dissected brains of age matched wild type *hALFY* and mutant *hALFY* transgenic pharates just prior to eclosing, and visualized their morphology using confocal microscopy. Unlike the normal morphology of brains of pharates expressing wild type *hALFY*, brains expressing mutant *hALFY* were 40–60% smaller in volume, denser, very fragile and malformed, and in some cases disintegrated during dissection (Fig 2C). Interestingly, while hALFY wild type brains displayed normal elongated neurons, in flies expressing mutant hALFY, clusters of disorganized cells containing aggregates of EGFP-labeled ALFY were evident (Fig 2C, white arrows). Although the expression of the mutant allele was ubiquitous, no clear effect was observed other than in brain and neuronal tissues.

Since the Drosophila eye is of neuronal origin and was previously used to explore phenotypes in mutants of the *ALFY* ortholog *Bluecheese* (*Bchs*), [20, 22] we used GMR-Gal4 promoter to drive gene expression in the eyes. While expression of the wild type *hALFY* C-terminal allele in the Drosophila eye had no effect on eye development, exogenous expression of the human mutant allele resulted in a severe rough eye phenotype (Fig 2B): the omatids were disorganized and of variable size and shape with many omatids fused together; the bristles were disorganized with irregular numbers stemming from in between omatids. The phenotype observed was evident immediately after eclosing.

### Functional in-vitro experiments

**Fig 3:**
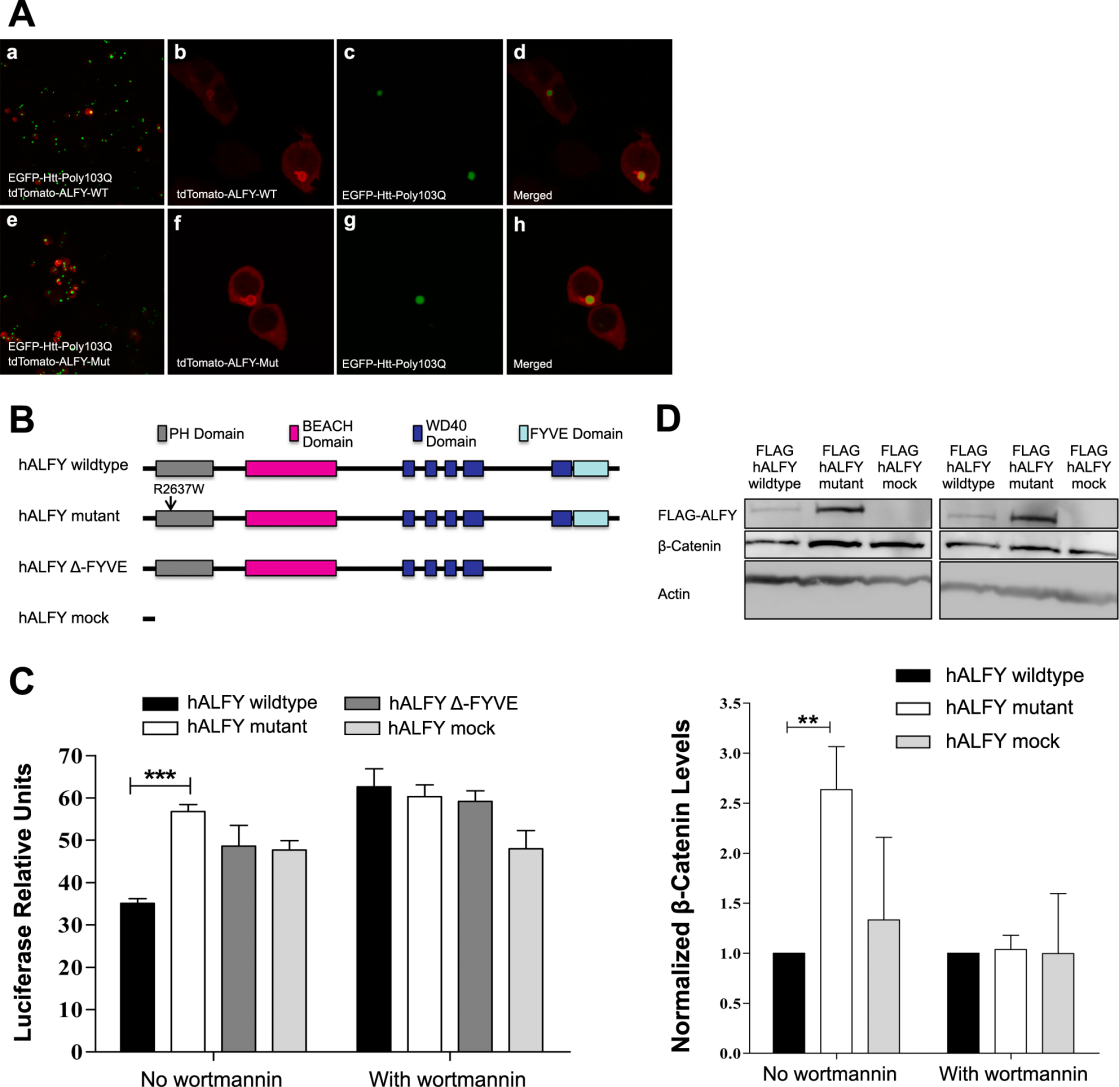
Htt-Poly103Q aggregate removal and Wnt signaling attenuation by *hALFY* protein. **A. Aggregate encapsulation and removal by hALFY.** HEk293T cells were co-transfected with EGFP-Htt-Poly103Q and tdTomato hALFY (wild type and mutant) (a-h). There was no significant difference in total amount of aggregates between wild type hALFY to mutant (a, e). Both wild type hALFY (b-d) and mutant hALFY (f-h) can identify and encapsulate aggregated Poly103Q. **B. Cloning of the hALFY constructs.** Schematic representation of the C-terminal end of *hALFY* constructs containing all functional domains of the protein. Four hALFY constructs were created: wild type, mutant, Δ-FYVE and mock tagged with N-terminal FLAG or tdTomato epitopes. **C. TOP Flash reporter assay demonstrates that wild type hALFY attenuates Wnt signaling.** Neuroblastoma cells were co-transfected with TOP Flash reporter assay constructs as well as the different hALFY plasmids (wild type, mutant, Δ-FYVE and hALFY-mock). Cells expressing wild type hALFY demonstrate a reduction of approximately 40% in luciferase expression relative to mutant hALFY and Δ-FYVE or mock controls (student t test, n = 3, P_value_ = 0.0005). Moreover, this attenuation is achieved probably in an autophagy dependent manner as could be seen by the addition of autophagy inhibitor wortmannin. **D. ALFY attenuates the canonical Wnt signaling pathway.** Cells over expressing the WT ALFY demonstrate lower levels (approximately 80% reduction, student t test, n = 3, P_value_ = 0.0028) of endogenous β-catenin.

To unravel the molecular pathway through which the *ALFY* mutation causes the disease phenotype, we tested mutant ALFY’s ability to exert its known function in aggregate removal. To that end, we transiently transfected HEK293T cells with the first exon of the huntingtin gene coupled with 103 PolyQ expansion repeats, tagged with an enhanced green fluorescent protein (EGFP tagged Htt-poly103Q) [19, 24]. Cells were co-transfected with plasmids expressing tdTomato-tagged constructs of either wild type or mutant hALFY C-terminus, which has previously been shown to harbor all the essential functional domains of the protein [19, 20, 25]. As seen in Fig 3A, the mutation had no effect on PolyQ aggregate encapsulation and removal by hALFY. We therefore set out to elucidate other, possibly novel autophagy-related functions of ALFY, which might unravel the molecular pathway through which the ALFY mutation causes the microcephaly phenotype.

Many of the known MCPH genes encode proteins that are associated with formation, stabilization and function of primary cilia [1, 5, 9]. Among their known functions, primary cilia mitigate Wnt signaling [26]. The Wnt signaling pathway, in turn, has been shown to be negatively regulated by autophagy-mediated degradation of disheveled [27]. The three human disheveled genes (DVL1, DVL2 and DVL3) encode hub proteins known to control Wnt signaling [28, 29]: upon Wnt signaling stimulation, DVL proteins, in their polymer form, recruit the β-catenin destruction complex to the membrane receptors LRP6 and Frizzled, releasing β-catenin, leading to its accumulation in the cytoplasm. β-catenin then shuttles to the cell nucleus, where it serves as a transcription factor activating transcription of Wnt signaling genes [30]. In contrast, ubiquitin-mediated autophagy of DVL proteins abrogates this process, attenuating Wnt signaling [27, 31]. The specific molecular pathway mediating DVL autophagy is yet unknown. We thus hypothesized that ALFY, a known master scaffold autophagy-related protein, might be the missing link, regulating DVL autophagy, thus controlling Wnt signaling.

### ALFY attenuates canonical Wnt signaling

To test whether hALFY (and its mutation) might affect Wnt signaling, we utilized the TOP Flash reporter assay: SH-SY5Y neuroblastoma cells were co-transfected with Renilla luciferase (normalizer construct) controlled by HSV TK promoter, and with firefly luciferase under the control of the TCEF/LEF promoter, a known reporter of Wnt signaling activation. Upon addition of Wnt3a conditioned medium to the cell culture, the Wnt pathway is activated, and its activity can be measured by the quantification of firefly luciferase in proportion to the control Renilla luciferase. In addition to the TOP Flash reporter assay constructs, the neuroblastoma cells were co-transfected with different hALFY constructs: wild type *hALFY*, mutant *hALFY*, and the negative controls *hALFY* Δ-*FYVE* and *hALFY-mock*, all tagged with either Flag or tdTomato. The constructs were generated using the *hALFY* wild type construct as template, as follows: hALFY mutant—by inserting a single point mutation recreating the exact p.R2637W substitution observed in our patients; hALFY Δ-FYVE, introducing a stop codon eliminating translation of the last of the five WD40 domains and the FYVE domain; hALFY-mock–inserting a premature stop codon eliminating all functional domains of the ALFY protein (Fig 3B, detailed in Methods).

As seen in Fig 3C, expression levels of firefly luciferase were ~40% lower in cells transfected with wild type ALFY in comparison with cells overexpressing hALFY mutant, hALFY Δ-FYVE or hALFY-mock. This ALFY-mediated attenuation of Wnt signaling was abrogated by the addition of wortmannin, a PI-3-kinase inhibitor, known to inhibit autophagy processes [19]. Thus, ALFY attenuates Wnt signaling, probably in an autophagy-dependent manner. Note that cells transfected with mutant hALFY exhibited slightly higher (albeit not statistically significant) luciferase levels compared to cells transfected with Δ-FYVE and mock controls, suggesting a possible dominant negative effect of the mutation.

We next set out test whether this ALFY-controlled Wnt signaling occurs via the canonical pathway. Activation of the canonical Wnt signaling pathway is known to result in higher levels of cytoplasmic β-catenin [30]. In line with the TOP Flash reporter assay data, β-catenin endogenous protein levels were lower in cells transfected with wild type hALFY as compared to cells expressing mutant hALFY (Fig 3D). Here too, introduction of the autophagy inhibitor wortmannin abrogated the differential effect seen (Fig 3C). These data confirm that ALFY regulates Wnt signaling most likely through an autophagy-mediated process, demonstrating that this occurs specifically through the canonical Wnt signaling pathway. The high levels of β-catenin in cells transfected with mutant hALFY in comparison to wild type hALFY further suggest a dominant negative effect of the mutation (Fig 3D).

### ALFY removes DVL3 aggregates

**Fig 4:**
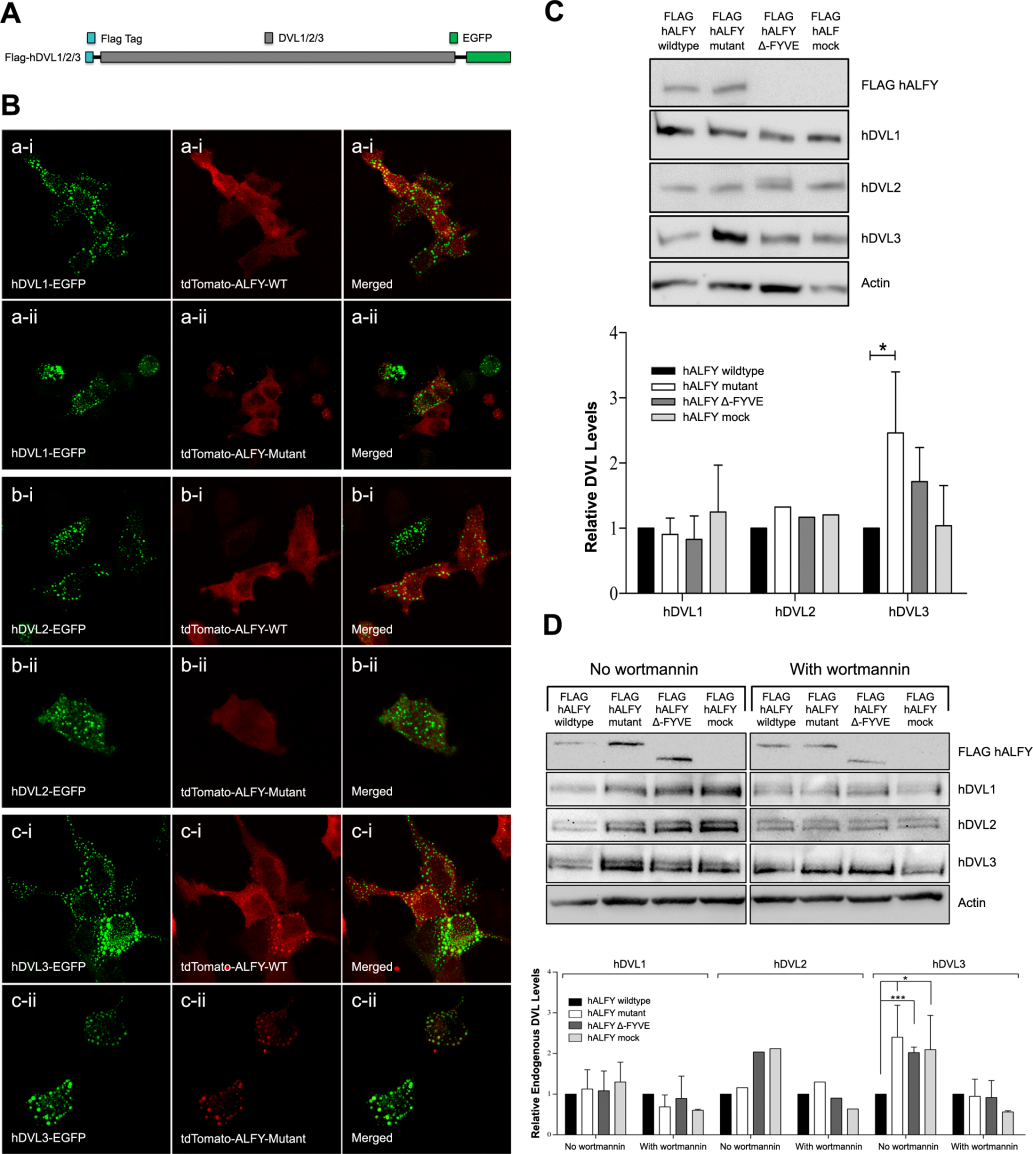
hALFY protein co-localizes with aggregated DVL3 and facilitates its removal. **A. Cloning of the different DVLs.** The three different *DVLs* were PCR-amplified from brain cDNA and cloned in frame with an N-terminal FLAG tag and a C-terminal EGFP. **B. Co-localization of hALFY with the different DVLs.** Neuroblastoma cells were co-transfected with the different DVLs (1, 2 & 3) as well as tdTomato hALFY wild type/mutant constructs. Both wild type and mutant hALFY co-localize specifically with DVL3 (c-i, c-ii) and not DVL1 (a-i, a-ii) or DVL2 (b-i,b-ii). Minimal co-localization with DVL1 was visible (a-i, a-ii), and could be due to the strong over expression. **C. Western blot analysis of DVL over expression.** Neuroblastoma cells were co-transfected with the different DVLs and FLAG hALFY (wild type, mutant, Δ-FYVE and mock). After 24 hours, cells were treated with cycloheximide to arrest further de-novo protein synthesis. WB analysis reveals that hALFY wild type removes aggregated DVL3 (student t test, n = 3, P_value_ = 0.04) yet not DVL1 or DVL2. DVL3 protein accumulated in hALFY mutant expressing cells. **D. Western blot analysis of endogenous DVLs.** Following transfection of the different FLAG hALFY constructs, neuroblastoma cells were incubated with Wnt3a conditioned medium for 8 hours and harvested for WB analysis. hALFY wild type specifically removed endogenous DVL3 aggregates, yet not DVL1 or DVL2. This effect was inhibited by wortmannin, indicating that hALFY removes DVL3 aggregates presumably in an autophagy dependent manner (student t test, n = 3, P_value_ = 0.03).

It has been previously shown that autophagy negatively regulates canonical Wnt signaling by promoting disheveled degradation [27]. Thus, we speculated that the ALFY autophagy-mediated attenuation of the Wnt signaling pathway that we identified might be mediated through disheveled degradation. To test this hypothesis, we transiently co-transfected neuroblastoma (SH-SY5Y) cells with constructs harboring human DVL1, DVL2 or DVL3 with a FLAG epitope tag in the N-terminus and EGFP in the C-terminus (Fig 4A). As expected, over-expression of the different DVL isoforms resulted in dynamic aggregate formation [32] of the respective DVL proteins, as could be seen in green puncta in confocal analysis (Fig 4B). We then repeated the above experiment, co-transfecting the cells also with either wild type or mutant human ALFY. As seen in Fig 4B, both wild type and mutant ALFY co-localized specifically with DVL3 rather than with DVL1 or DVL2 (minimal co-localization with DVL1 was seen, but was significantly lesser than with DVL3 and might be secondary to the massive overexpression levels). As DVL proteins are continuously generated, to test their removal we repeated the above experiment with the addition of cycloheximide 24 hours following transfection, preventing further de-novo translation of DVL proteins. Amounts of DVL proteins were assessed by western blotting using antibodies to the FLAG tag epitope of the DVLs. As seen in Fig 4C, in the presence of wild type hALFY, DVL3 protein levels were lower (though not significantly) than in the presence of the Δ-FYVE or mock controls lacking hALFY’s crucial functional domains. In line with likely dominant negative effect of the mutation, this effect was even more apparent and significant when comparing the effect of wild type hALFY to that of mutant hALFY, with significantly higher DVL3 levels in the presence of mutant hALFY. Thus, hALFY acts in DVL3 aggregate removal. In contrast, there was no visible effect of hALFY on aggregate removal of DVL1 and DVL2 (Fig 4C). We repeated the experiment exploring the effect of hALFY on endogenous DVL protein levels. As shown in Fig 4D, the levels of endogenous DVL3 were significantly lower in cells over-expressing wild type hALFY in contrast with cells over-expressing either mutant, Δ-FYVE or mock negative control hALFY constructs. Moreover, this effect of wild type hALFY was abolished in the presence of Wortmannin. In line with the previous results, hALFY over-expression did not significantly alter levels of DVL1 or DVL2. Thus, our data taken together with the known role of ALFY in autophagy, demonstrate specific targeting and autophagy-mediated removal of DVL3 by hALFY.

## Discussion

Through linkage analysis and whole exome sequencing, we demonstrated that dominantly inherited human congenital microcephaly with mild to moderate intellectual disability is caused by a mutation in hALFY (termed also *WDFY3*). *hALFY*, encoding an autophagy master scaffold protein, is ubiquitously expressed and its expression levels in mouse tissues ranges from low (heart, kidney, lungs, skeletal muscle and pancreas) to extremely high levels (brain, spleen) [19]. In mice, expression of the ALFY homolog *wdfy3* is maintained in the neocortex of the developing embryo [33], specifically in dividing neuronal progenitor cells (RGCs), and is required for a specific subset of progenitor divisions [34]; its Drosophila homolog, *Bchs*, is expressed in the outer cortical regions of the nervous system and in the ommatidial cluster of the developing eye after neuronal differentiation [22]. It is noteworthy that this expression profile of ALFY is similar to that of other microcephaly genes [5, 8, 9, 14, 15, 35].

Null mutants of ALFY homologs have been generated and studied both in flies and in mice: *Bchs* knockout files develop normally and display normal motor, feeding, and grooming behaviors [22]. However, the life span of those mutants is 40–50% shorter than that of wild type flies. Furthermore, the mutant flies display a progressive neurodegenerative phenotype and reduction of approximately 40% in brain volume [22]. Mice with null mutations in *wdfy3* have also been recently generated [34]. While heterozygous mutant mice show no abnormal phenotype, homozygous mutants are embryonic lethal, with a clear brain phenotype: the cerebral cortex is visibly thinner and tangentially longer compared to wild type controls. The proliferative regions of the cortical ventricular and subventricular zones as well as the intermediate zone are thinner in mutants, while the cortical plate and marginal zone are not affected in thickness [34]. The tangential expansion but lateral thinning of the neocortical neuroepithelial seen in those mice, suggest an imbalance in the mode of cortical progenitor cell divisions, favoring proliferative at the expense of differentiative divisions [34].

Although the embryonic phenotype of the mouse mutants has been well studied, the molecular mechanisms underlying normal and abnormal ALFY-related neurodevelopment are yet unknown. To address this issue, we extended our studies, exploring the effects of human wild type and mutant ALFY in the drosophila model system and in vitro experiments. First, we verified the dominant effect of the human ALFY mutation through Drosophila experiments: while transgenic flies expressing the human wild type ALFY had no abnormal phenotype, transgenic flies expressing human mutant ALFY driven by a ubiquitous actin promoter were lethal and had an evident neuro-specific phenotype with smaller brain size, similar to that seen in *bchs* null mutant flies [22]. As no abnormalities were evident in the transgenic flies aside from the brain phenotype, it is likely that the late embryonic lethality was secondary to the neurological defect. It is noteworthy that the brain-specific microcephaly phenotype seen in flies expressing the human mutant ALFY strikingly recapitulates the dominant phenotype seen in our patients, in line with the extreme conservation of ALFY throughout evolution.

The dominant effect of the hALFY mutation was further demonstrated through studies of the Drosophila eye, a well-studied model system of neurodevelopment [36, 37] Eye-specific GMR-promoter driven expression of mutant hALFY generated a rough eye phenotype, as compared with no abnormal phenotype in flies expressing wild type hALFY. The Drosophila rough eye phenotype, disrupting the regular ommatidia arrangement, has been demonstrated in mutations in various genes controlling neurodevelopment, including, for instance, lgl1 [38], whose mouse null mutants demonstrate failure of neural progenitor cells to exit the cell cycle and differentiate, and instead, continue to proliferate and die by apoptosis [39]. Thus, our data suggest a possible role of ALFY in controlling proper transition of neuronal progenitor cells from proliferation to differentiation. This point will be dealt with later in the discussion.

Since ALFY is an autophagy-mediating scaffold protein, capable of removing Htt-Poly103Q aggregates [18, 19, 21], we tested whether this function is affected by the mutation. As seen in Fig 3A, mutant hALFY was as effective as wild type hALFY in Poly103Q aggregate removal. This is in line with the disease phenotype being one of primary congenital microcephaly rather than a progressive neurodegenerative disease. However, the question of molecular mechanisms through which the ALFY mutation exerts its effect remained unclear.

*ALFY* encodes a large nuclear protein, which shuttles to the cytoplasm upon formation of protein aggregates: upon generation of aggregates in the cytoplasm, they are ubiquinated and bound by P62. P62, a nuclear protein, is thought to bind ALFY through its PH-BEACH domain and shuttles the protein complex to the ubiquinated aggregates. ALFY then binds through its WD40 domains the ATG complex of autophagy proteins, and through its FYVE domain LC3-bound membranes, thus putting together autophagosomes. This is followed by fusion of autophagosomes with lysosomes to generate autolysosomes, where aggregated proteins are disintegrated [18, 40]. Thus, ALFY serves as a scaffold protein recruiting the autophagy machinery to generate the autophagosome, enabling degradation of cytosolic protein aggregates.

All genes associated to date with human MCPH are associated with processes of cell cycle control, such as DNA replication, primary cilia formation and stabilization, and centriole duplication [5, 8, 9, 15, 35, 41–54]. Such processes govern properly timed transition from symmetric to asymmetric cell division in the developing brain. Taking into consideration ALFY’s role in autophagy and the known association of MCPH with defects in cell cycle regulation, we set out to elucidate a novel, yet unraveled, function of ALFY that might integrate those two processes. The Wnt signaling pathway is one of the major molecular pathways controlling cell division and the transition between symmetrical and non-symmetrical cell division [26, 55–59]. In fact, proliferating cells are known to demonstrate high Wnt signaling activity [56, 58, 60]. It has previously been shown that autophagy negatively regulates the Wnt signaling pathway through degradation of disheveled (DVL) proteins, hub proteins controlling Wnt signaling [27]. We thus hypothesized that ALFY might be the missing link connecting the Wnt signaling pathway and DVL autophagy. First, using the TOP Flash assay we showed that ALFY normally attenuates Wnt signaling, an effect abrogated by either the ALFY mutation seen in the patients, or null mutations introduced such that ablate its crucial domains. Furthermore, through studies of β-catenin levels, we showed that the effect of ALFY is specifically in controlling the canonical Wnt signaling pathway. Repeating the experiments with and without a known autophagy inhibitor, we showed that this ALFY-mediated regulation of the canonical Wnt signaling is most likely achieved through autophagy. Finally, we showed that ALFY co-localizes with DVL3 and facilitates the removal of DVL3 aggregates from the cytoplasm. Both co-localization and aggregate removal experiments demonstrated that the effect was very specific: ALFY did not co-localize with or act in removal of aggregates of DVL1 and DVL2. Thus, we show a novel molecular mechanism of regulation of the canonical Wnt signaling pathway through ALFY-mediated regulation of DVL3 aggregate removal.

Our data heavily suggest that the hALFY mutation acts through a dominant negative effect rather than haploinsufficiency: over-expression of the mutant hALFY (and not wild type hALFY) was sufficient to generate a Drosophila phenotype regardless of the endogenous Bchs protein, practically ruling out haploinsufficiency as a mechanism through which the mutation acts. In the cell culture experiments, Wnt signaling activity, as exhibited by both TOP Flash luciferase activity and beta-catenin levels, was consistently slightly higher in cells expressing mutant hALFY than in those expressing the Δ-FYVE or hALFY-mock control lacking hALFY’s crucial functional domains. This, again, suggests a likely dominant negative rather than dosage effect. The same is true also for the effects on DVL3 aggregate removal further supporting our claim.

**Fig 5:**
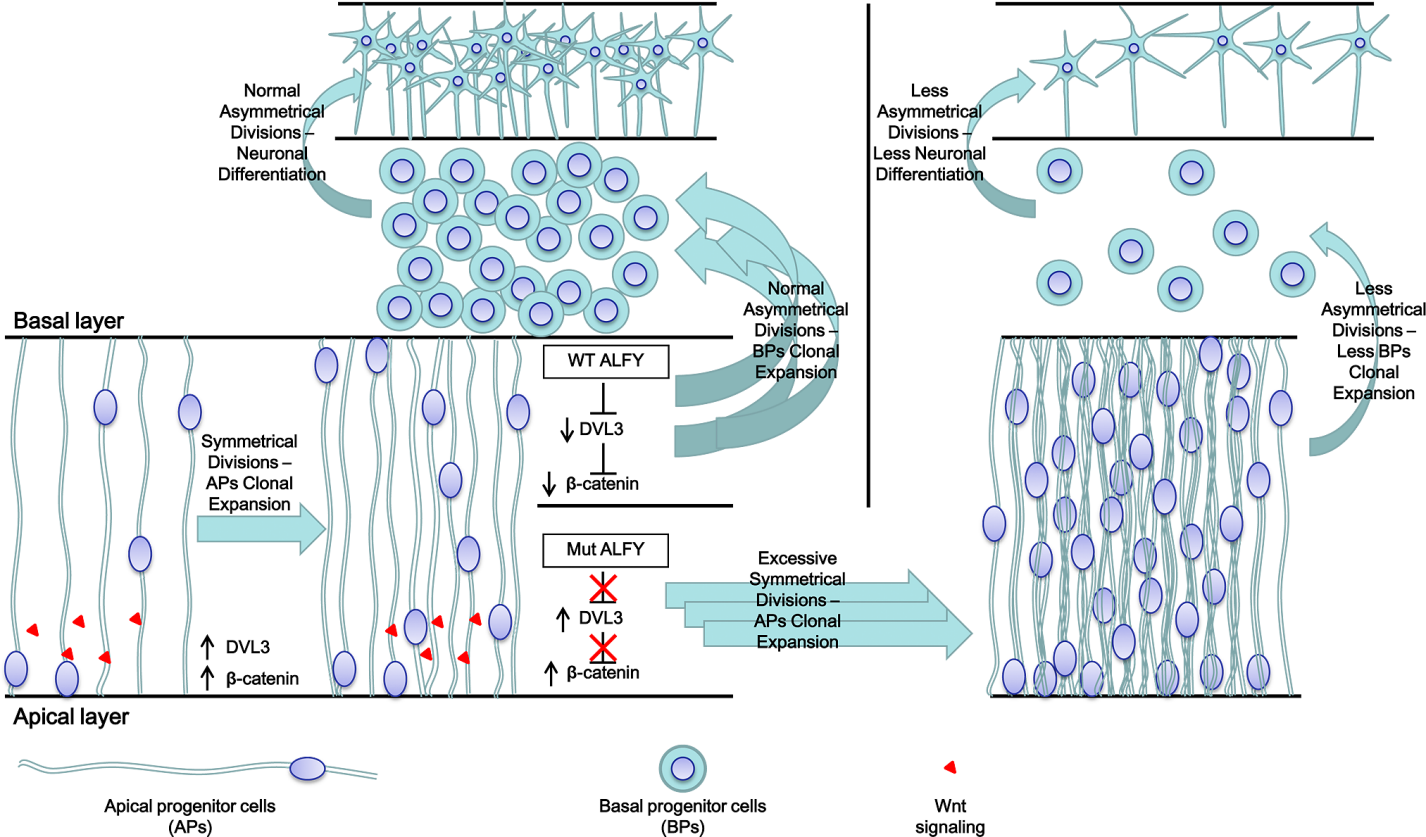
Proposed model—ALFY mediates proper brain development through DVL3-autophagy-mediated precise control of the canonical Wnt signaling pathway. Proper embryonic brain development and size are achieved through clonal expansion of apical progenitors (APs), followed by expansion of basal progenitors (BPs). The first step is clonal expansion of the AP cell layer: AP cells undergo a series of Wnt signaling-induced proliferative symmetrical divisions. Due to Wnt signaling activation, DVL3 levels increase and the β-catenin destruction complex is recruited to the membrane, preventing it from phosphorylating β-catenin, leading to accumulation of β-catenin in the cytoplasm and eventually in the nucleus. This triggers activation of Wnt signaling-specific genes that allow the APs to further proliferate and maintain their progenitor identity, generating the first clonal expansion in the developing brain. Once there is sufficient number of AP cells, ALFY attenuates Wnt signaling by autophagy-mediated removal of DVL3, allowing the β-catenin destruction complex to phosphorylate β-catenin, resulting in its removal from the cytoplasm. This attenuation of the Wnt signaling enables the cells to start asymmetric differentiative divisions, generating the second critical layer in the developing brain, the BPs. The BPs will continue to proliferate and eventually differentiate to generate neuronal cells. In the absence of appropriate ALFY activity (right panel) DVL3 levels remain high, resulting in elevated β-catenin levels and high activation of the Wnt signaling pathway. This lack of attenuation of Wnt signaling prevents proper generation of the BP cell layer and therefore reduces the generation of differentiated neurons. We believe that this locks the AP cells in successive rounds of symmetrical proliferative divisions, further expanding their population at the expense of generating the crucial BP cell layer, resulting in major reduction of BPs. Since the AP layer is confined in space, this expansion of APs cells is limited and will not compensate for the lack of BPs. It is the BP cell layer that demonstrates the most significant difference between primates and rodents and is believed to be responsible for determining the final size of the primate brain [3]. Thus, lack of BP cells in the developing brain eventually leads to reduced number of neurons and microcephaly.

Regarding the mechanism through which the ALFY mutation causes primary microcephaly, taking all our data together with that of the previously described Wdfy3 null mutants, we suggest the following model (Fig 5): during normal brain development the apical progenitor (APs) cell layer undergoes a series of symmetrical proliferative divisions, triggered by Wnt signaling. This cell proliferation constitutes the initial clonal expansion of cortical brain cells. At this point, ALFY which is expressed in this layer of proliferating RGCs [30], attenuates Wnt signaling through DVL3 aggregate removal, leading to inactivation of the Wnt signaling resulting in transition to asymmetrical differentiative cell division, generating the basal progenitor (BPs) cell layer. Expansion of this second cell layer is essential in primate brain development and cortex expansion [3]. This BPs layer will eventually differentiate to form functional neurons. In the mutant ALFY this attenuation is abrogated. Therefore, Wnt signaling proceeds unharmed, "locking" the AP cells in further consecutive rounds of symmetrical cell divisions, at the expense of formation of BPs, eventually resulting in fewer neurons and thus microcephaly. This is in line with the 50% reduction in BP cell number and increase in proliferative symmetrical division at the expense of asymmetrical divisions, resulting in tangential expansion and radial thinning of the cortex seen in Wdfy3 null mutant mice[34]. Since the AP cell layer is confined in space, this expansion of APs is limited and will not compensate for the lack of crucial BP cells. It is the BP cell layer that demonstrates the most significant difference between primates and rodents and is believed to be responsible for determining the final size of the primate brain [3, 61]. Our data together with the known functional roles of ALFY, suggest a novel, previously undescribed role for autophagy in neurodevelopmental processes and proper brain development, and demonstrate that defective autophagy-related processes can cause MCPH.

## Methods

### Ethics statement

The study was approved by the Soroka Medical Center institutional review board (approval number 5071G) and the Israel Ministry of Health National Helsinki committee (approval number 920100319). Written informed consent, per the study approved protocol, was obtained from all individuals studied or their legal guardians.

### Research subjects

The study protocol was approved by Soroka Medical Center institutional review board. Informed consent was provided by all participants or their legal guardians.

### Genetic analysis

Genome-wide linkage analysis of six family members (five affected, one unaffected) was done (ABI PRISM Linkage Mapping Set MD10, Applied Biosystems) and analyzed using GeneScan software, as previously described.[62] Fine mapping analyzing nine affected and seven unaffected individuals was carried out as previously described [62] using polymorphic markers indicated in the family pedigree (Fig 1A). PCR products were separated on 6% polyacrylamide gel and visualized by silver staining, and haplotypes were manually constructed and analyzed. Maximal LOD score was calculated using SUPERLINK.[62] Whole exome sequencing was performed as previously described [63] for individual II:4 (Fig 1A) and data was analyzed using QIAGEN’s Ingenuity Variant Analysis software (http://www.qiagen.com/ingenuity) from QIAGEN Redwood City. Using their filtering cascade, we excluded variants observed with an allele frequency ≥1.0% of the genomes in the 1000 genomes project, National Heart, Lung, and Blood Institute (NHLBI), Exome Sequencing Project (ESP) or the Allele Frequency Community. In addition, we excluded variants that appeared in our in-house whole exome sequencing database of 100 Bedouin control samples. Furthermore, we kept variants that are predicted to have a deleterious effect upon protein coding sequences (eg, frameshift, in-frame indel, stop codon change, missense or predicted to disrupt splicing by MaxEntScan) and variants that are experimentally observed to be associated with a phenotype: pathogenic, possibly pathogenic or disease-associated according to the Human Gene Mutation Database (HGMD).

### Variant screening

Screening for the *CXCL11* 3-nucleotide deletion mutation was done using PCR amplification followed by separation on a 6% polyacrylamide gel and visualization by silver staining (details available upon request). For the *ALFY* mutation, screening was done using BtsCI restriction analysis of PCR amplicons (Forward primer 5’-CCCTCCCATATCTTCCCATAA-3’; Reverse 5'-GCCGCAGATTAACTTCTTGA-3’), demonstrating a 240 bp wild type allele versus 100 bp and 140 bp fragments for the mutant allele.

### Cloning of Drosophila constructs

All clones were generated with a modified pUAST vector which contains an EGFP fragment and an attb1 site which enables site-directed insertion of the constructs. Plasmids containing the C-Terminal part of hALFY, which contains all functional protein domains (PH-BEACH, WD40 and FYVE domain), were a kind gift from Prof. Anne Simonsen and were used as template for PCR amplification. Primers were designed to match *ALFY* gene sequence, as well as to contain a homologous sequence for the pUAST plasmid at the 5' end and an EGFP homology sequence at the 3' end (Forward primer 5'-CTGCCAAGAAGTAATTATTGAATACAAGAAGAGAACTCTGAATAGGGAATTGGGAATTCCAAAATGTTAACAGGATCAAGAAGGAATC-3' and reverse primer 5'-GCTCGACCAGGATGGGCACCACCCCGGTGAACAGCTCCTCGCCCTTGCTCACCATGAATTCTCTACAACAATTTCGAGGCCCATCTTC-3'). The 3756 bp PCR amplicon was inserted into the pUAST vector using homologous recombination in yeast. The PCR product was cloned in frame with EGFP sequence at the 3' end of *hALFY* to generate ALFY-EGFP fusion protein. Transgenic flies were generated by Genetic Services Inc. (Biotech Center, Boston, Massachusetts, USA) in a site specific manner. To reduce variability of expression due to position effects, the final wild type and mutant constructs were inserted at the same attp2 site on the 3rd chromosome, which is known for its strong and stable expression levels.[64] One line for the wild type and two lines for the mutant construct were generated. The different lines were balanced to form a balanced lethal stock.

### hALFY constructs

Constructs containing the C-terminal part of human *ALFY* gene as well as Htt-Poly103Q were a kind gift from Prof. Simonsen. Tagged versions of hALFY gene (N-terminal FLAG tag, N-terminal tdTomato and a C-terminal EGFP tag), containing all functional domains, spanning 2285–3526 amino acids of ALFY were obtained and used as a template for generating the different constructs used in our experiments. The same technique was employed with different primers to generate the different constructs. We performed PCR amplification using modified back to back primers containing the desired mutations, followed with DpnI treatment to remove template plasmid DNA. Finally, the mutated constructs were validated with Sanger sequencing and restriction analysis. We performed this mutagenesis on all available constructs (FLAG, tdTomato and EGFP). To generate the hALFY mutant (R2637W) constructs we inserted a single point mutation, replicating the mutated Arg to Trp substitution seen in our patients. To that end we used modified back to back primers containing the mutation (WDFY3-pMut-F-5'-CTCTGGAGATGGAtGGAATTACCTC-3', WDFY3-pMut-R-5'-GAGGTAATTCCaTCCATCTCCAGAG-3'). The ALFY Δ-FYVE constructs were generated by inserting a premature stop codon prior to the last WD40 domain with mutated primers (ALFY Cter-Δ-FYVE-F-5'-GGAGCCAGCAGATCATCTGaTGCTGCATGTCGGAGATG-3', ALFY Cter-Δ-FYVE-R-5'-CATCTCCGACATGCAGCAtCAGATGATCTGCTGGCTCC-3'), resulting in truncated protein lacking the crucial WD40 and FYVE domains. Finally, the ALFY-mock constructs were generated by inserting a premature stop mutation prior to the PH-BEACH domain using mutated primers (ALFY Cter-mock-F-5'- GAAGAGCCGTAAGTTAacACAGTAAAGAG-3', ALFY Cter-mock-R-5'- CTCTTTACTGTgtTAACTTACGGCTCTTC-3'). The inserted mutation results in a 133 amino acid truncated protein (of the 1226 amino acids full length ALFY) lacking all known functional domains.

### Htt-Poly103Q and DVL constructs

The different *DVL* genes were PCR amplified from brain cDNA library to obtain the full length constructs (DVL1-F–5'-GCGGAGACCAAGATTATCTACC-3', Acc65I-DVL1-R–5'-GGTACCtCATGATGTCCACGAAGAACTCGCAG-3', DVL2-F-5'-GCGGGTAGCAGCACTGGGG-3', Acc65I-DVL2-R- GGTACCtCATAACATCCACAAAGAACTC-3', DVL3-F-5'-GGCGAGACCAAGATCATCTACCAC-3', Acc65I-DVL3-R-5'-GGTACCtCATCACATCCACAAAGAACTC-3') followed by a second round of PCR using modified primers to insert a FLAG epitope at the N terminal end of the protein as well as Acc65I restriction sites (Acc65I-Flag-DVL1-F–5'-GTACCATGgattacaaggatgacgatgacaagGCGGAGACCAAGATTATCTACC-3', Acc65I-Flag-DVL2-F-5'-GGTACCATGgattacaaggatgacgatgacaagGCGGGTAGCAGCACTGGGG-3', Acc65I-DVL3-Flag-F-5'-GGTACCATGgattacaaggatgacgatgacaagGGCGAGACCAAGATCATCTACCAC-3'). The modified PCR fragments were cloned into a pEGFP-N2 vector in frame with a C terminal EGFP protein using Acc65I restriction enzyme. Further information regarding the different cloning will be delivered upon request.

### Wnt3a conditioned medium

Wnt3a conditioned medium was obtained by collecting growth medium from mouse fibroblast secreting the active form of Wnt3a glycoprotein (ATCC CRL-2647) according to the manufacturer protocol. In brief: cells were split and grown in T-75 flasks in normal DMEM medium (without G418) for 4 days and growth medium was collected and filter sterilized to achieve the first batch of medium. The medium was replaced and cells were grown for additional 2–3 days and again medium was collected and filter sterilized to achieve the second batch. The two batches were than mixed to generate the Wnt3a conditioned medium.

### Wnt signaling measurements

To assess the activation of the Wnt signaling we utilized the standard, largely accepted TOP Flash reporter assay. Twelve hours prior transfection SH-SY5Y neuroblastoma cells were plated in 24-well tissue plates to approximately 60–70% confluence 12 hours prior to transfection. Cells were then transfected with lipofectamine2000 (Invitrogen) according to manufacturer protocol with 400 ng of TOP flash plasmids (Millipore), 400 ng the different FLAG-ALFY constructs and 50 ng of internal normalizer pGL4.74 [hRluc/TK] vector (Promega). The experiments were repeated with the negative control FOP Flash (Millipore) to verify the activity of the TOP Flash reporter system and indeed FOP Flash luciferase expression levels were zero. Autophagy inhibition was achieved by adding wortmannin (0.2μM final concentration, ab120148 –abcam) for the entire duration of the experiment. Following transfection, cells were incubated with the plasmid mixture over night and the following day the growth medium was replaced with Wnt3a conditioned medium (with or without wortmannin) for a period of 24 hours. Finally, after 24 hours of induction, the cells were harvested, lysed and the luciferase reporter gene assay was conducted (Dual-Luciferase Reporter Assay System, Promega, E1910). Each transfection was repeated 3 times (3xALFY wild type, 3xALFY Mutant etc.) and each transfected well was measured 3 times for luciferase activity. Average values per transfected well were calculated to obtain a precise measurement of Wnt signaling activity per single well which was later used to calculate the average value for each experiment. Luciferase activity was measured using TECAN infinite M200 instrument.

### Cell fixation and confocal microscopy

#### Co-localization experiments

SH-SY5Y neuroblastoma cells were plated to a confluency of approximately 60–70% on coverslips in a 12-well tissue plates 12 hours prior to transfection. The cells were co-transfected with 500 ng of the different DVLs (1, 2 or 3) and 500 ng of the different tdTomato-ALFY constructs (wild type, mutant or Δ-FYVE). Cells were left to incubate with the plasmid mix overnight and the medium was replaced the following morning with complete growth medium. Fixation was performed 24 hours after transfection. Coverslips were rinsed with PBS and fixed in 4% paraformaldehyde (Santa Cruz, Santa Cruz, CA, http://www.scbt.com) for 20 minutes, washed twice with PBS, permeabilized using 0.1% Triton in PBS for 15 minutes, and washed three times with PBS to remove detergent. Finally, coverslips were mounted using Vectashield with DAPI (Vector Labs, Peterborough, UK) and visualized using confocal microscopy.

### WB analysis

Protein lysates were heated for 10 min in 95°c and loaded onto 8% polyacrylamide gel. Following electrophoresis at 150V for 1.5 hours, proteins were transferred to nitrocellulose membranes for 1 hour at 300 mA. The nitrocellulose membranes were blocked by incubation in TTBS (0.02M Tris, pH7.5, 0.15M NaCl, 0.9mM Tween 20 –Bio Lab) containing 3% BSA for 1 hour at room temperature. The blocked membranes were incubated for 1 hour at room temperature with primary antibodies, followed by 3 TTBS washes 10 min each. The membranes were then incubated with the appropriate secondary antibodies for 1 hour at room temperature, washed 3 times in TTBS and visualized using ChemiDoc MP imaging system (Bio Rad). Antibody binding was visualized using an enhanced chemiluminescence detection kit (SuperSignal West Pico chemiluminescent substrate–Thermo scientific). All experiments were repeated several times and were all normalized to wild type hALFY.

#### DVLs overexpression

SH-SY5Y neuroblastoma cells were transfected as described above in the co-localization experiments; however, for these experiments we used the FLAG-ALFY constructs instead tdTomato-ALFY. After overnight incubation of the plasmid mix, the medium was replaced with complete growth medium supplemented with cycloheximide (30 μg/ml—TOKU-E) for 10 hours. Once the incubation was completed, cells were lysed with protein lysis buffer and collected for further analysis. Primary antibodies used for this experiment: mouse anti-FLAG (1:3000-F1804, Sigma-Aldrich), HRP anti-actin (1:6000-sc-1616, Santa Cruz). Secondary antibodies: HRP (Goat) anti-mouse (1:10000-sc-2005, Santa Cruz).

#### Endogenous expression

For endogenous β-catenin, DVL1, DVL2 and DVL3 level detection, SH-SY5Y cells were plated in 24-well tissue plates and transfected with 400ng FLAG-ALFY constructs (wild type, mutant, Δ-FYVE and mock). After an ON incubation with the plasmid mix, cell's medium was replaced with Wnt3a conditioned medium for 8 hours. Total protein lysates were collected for further analysis. Primary antibodies used for this experiment: Mouse anti-FLAG (1:3000-Sigma-Aldrich), Rabbit anti-DVL1 (1:1500-D3570, Sigma-Aldrich), Rabbit anti-DVL2 (1:10000-D1321, Sigma-Aldrich), Rabbit anti-DVL3 (1:6000-SAB4200008, Sigma-Aldrich), Mouse anti-DVL3 (1:500-sc-377289, Santa Cruz), Rabbit anti-β-catenin (1:7000-ab32572, abcam), HRP (Goat) anti-Actin (1:6000-sc-1616, Santa Cruz). Secondary antibodies: HRP (Goat) anti-mouse (1:10000—sc-2005, Santa Cruz), HRP (Donkey) anti-rabbit (1:30000 –ab97085, abcam).

### Fly stocks

Transgenic flies for both wild type and mutant *hALFY* were generated (Genetic Services Inc.) and *F1* progeny were analyzed for eye phenotype in terms of pigmentation, size, shape and surface texture as well as for neuronal brain phenotype. For each of the experiments, one line for the wild type allele and two different lines for the mutant alleles were generated, with similar results. All fly lines were cultured and maintained on standard cornmeal/agar medium at 25°c. Actin- and GMR-Gal4 driver strains were obtained from Bloomington stock center and used to drive ubiquitous and eye expression of the ALFY constructs, respectively [65].

### Microscopy

Representative digital eye images were taken with a Leica 205C dissection microscope and Leica DSC290 HD camera system. In addition, samples were examined with a JEOL JSM 7400F scanning electron microscope (SEM). Brains were analyzed using confocal microscope and Z stack images of the brains were taken using bright field and 488nm filter.

### Brain dissection and volume calculation

Flies were anesthetized with carbon dioxide gas, rinsed once in 70% ethanol, twice in S2 media (Schneider’s Insect Medium, Sigma), submerged in cold S2 media on a dish, and dissected with Dumont #5 stainless steel forceps. Once the brains were exposed, membranes, trachea, and other tissues were removed, taking care not to touch the brain as was demonstrated in a video of the dissection procedure http://www.janelia.org/team-project/fly-light#5064.[66] The brains were transferred to 4% paraformaldehyde in PBS medium for 20 min followed by 2 washes in PBS and were mounted onto slides containing Vectashield mounting medium without DAPI. Drosophila brain volume was evaluated using the EGFP Z stack images obtained before. Using Icy software,[67] we selected a representative image from each Z stack acquired, which contained the largest part of the brain in the stack. The brain area of that stack was then calculated using the EGFP pixels to determine brain borders. Once brain size was evaluated, the following formula was used to evaluate brain volume:

$$Volume\;({mm}^{3})=\left(Area\;of\;Brain\;({mm}^{2})\right)*(Number\;of\;Z\;stacks)*(Z\;stack\;interval\;(mm))$$

All stacks were measured at 5μm (0.005mm) intervals, analyzing brains of 18 wild type *hALFY* and 31 mutant *hALFY* transgenic flies. The values obtained are a close approximation of the actual brain volumes; the process was performed in similar manner for both wild type and mutants.
